# Learning-Behavioral Affordances in German Textbooks: Sustainability-Oriented Intercultural Competence Development in China

**DOI:** 10.3390/bs16061028

**Published:** 2026-06-19

**Authors:** Chenxi Li, Enuo Wang

**Affiliations:** 1 School of Foreign Languages and Literatures, Lanzhou University, Lanzhou 730000, China; lichx@lzu.edu.cn; 2 School of Foreign Languages, East China Normal University, Shanghai 200050, China

**Keywords:** learning behavior, behavioral affordances, competence model, intercultural competence, sustainability-oriented intercultural learning, German-language education, China

## Abstract

This study examines how German textbooks provide learning-behavioral affordances for sustainability-oriented intercultural competence development. Drawing on Klieme’s competence-model logic, ESD, intercultural competence research, learning behavior theory, and affordance theory, it treats “sustainable intercultural competence” not as a standardized construct but as a working shorthand for the sustainability-oriented development of intercultural competence. Methodologically, the study adopts a directed qualitative content analysis supplemented by descriptive frequency aggregation. All 37 units across the four volumes of *Meilenstein* were coded on a 0–2 scale across three affordance dimensions: cognitive-understanding affordance, reflective value-judgment affordance, and interaction-action affordance. The findings show that the series provides substantial but uneven affordances. Interaction-action received the highest aggregated score, followed by cognitive-understanding, whereas reflective value-judgment remained substantially lower. Units on family, identity, sustainability, and civic engagement offer the most balanced affordance structures, whereas everyday practical units privilege communicative action and disciplinary units privilege cognitive understanding. The study argues that textbook-based intercultural learning should be examined not only through topic inclusion but also through how texts, prompts, and tasks organize opportunities for comparison, reflection, judgment, negotiation, and action.

## 1. Introduction

### 1.1. Background and Rationale

In foreign-language education, intercultural competence is not only a body of cultural knowledge or a set of communicative outcomes but also a process of learning behavior. Learners develop intercultural competence by noticing cultural differences, comparing perspectives, reflecting on values, regulating assumptions, negotiating meanings, and participating in communicative tasks. From this perspective, textbooks are not merely carriers of cultural content; they structure the behavioral opportunities through which learners are invited to practice intercultural understanding, reflection, and action.

Sustainable development has become a key reference point in contemporary education because it connects present development with future responsibility, ecological limits, social justice, and economic life (Solomon, 2023). In education, this broader orientation has taken shape in Education for Sustainable Development (ESD), which seeks to equip learners with the knowledge, values, and skills needed for responsible action (Dawe et al., 2005). Foreign-language education belongs in this discussion because sustainability is also discursive, cultural, and social. Language learning can help learners understand how local and global problems are framed, negotiated, and acted upon through discourse (Ge et al., 2023; Stibbe, 2008). Recent behavioral sciences research further suggests that intercultural communicative skills are associated with sustained language-learning behavior among Chinese college students (Yang et al., 2024).

The Chinese context is especially relevant here. Although English remains dominant, the sustainable development of languages other than English (LOTEs) has become increasingly important in Chinese language policy and curriculum reform (Ge et al., 2023). German remains a major LOTE in China, and German-language education offers a useful site for examining how sustainability-oriented aims are translated into teaching materials and classroom practice. This relevance is reinforced by recent behavioral sciences research showing that intercultural communication competence and intergroup attitudes predict Chinese students’ willingness to form intercultural friendships with international students (Tang & Zhang, 2023).

A central concept in foreign-language education, especially in German as a foreign language, is intercultural competence, understood here as the ability to understand cultural difference and communicate appropriately and effectively across cultures (Byram, 1997). Work on languaculture, and intercultural dialogue has shown that culture is not a supplementary background to language learning, but part of its core, and that cultural learning should avoid static or nationally closed views of culture (Byram & Wagner, 2018; Kramsch, 1993; Risager, 2005). ESD-oriented research adds another layer by drawing attention to sustainability-related awareness, responsibility, and action in language education. What remains less clear is how intercultural competence, ESD, and learning behavior can be translated into an operational framework for textbook analysis. Klieme’s competence-model logic is useful here because it explains how broad educational aims can be specified through domain-related competence dimensions and operational indicators (Klieme et al., 2003).

The same problem appears clearly in textbook research. Textbooks remain one of the main mediators between educational aims and classroom practice. They do not simply provide linguistic input. They also frame cultural perspectives, organize learning tasks, and shape which social meanings and forms of action become pedagogically salient. From an affordance-oriented perspective, the key question is not only what intercultural or sustainability-related content textbooks contain but what kinds of learning actions their texts, prompts, and tasks make possible. Existing scholarship has more often examined sustainability-related themes and general intercultural content separately than it has asked how textbooks transform such content into learning-behavioral affordances. This is particularly true of German-language textbooks used in China. Little research has examined how such materials guide learners from intercultural knowledge input toward reflective value judgment and action-oriented communication (Aronin & Singleton, 2012; Nguyen, 2025). This creates a specific research gap: although intercultural competence, ESD, and textbook analysis have each been studied extensively, less is known about how German textbooks used in China transform sustainability-oriented intercultural aims into concrete learning-behavioral affordances.

### 1.2. Klieme-Informed Analytical Framework

Intercultural competence is the immediate theoretical point of departure for this study, but the formal logic of the analytical framework is derived from Klieme’s competence-model approach. Klieme’s framework explains how broad educational aims can be translated into domain-specific competence dimensions, developmental levels, and operationalizable requirements (Klieme et al., 2003). This logic is central here: ESD and intercultural learning define the educational orientation; cognitive-understanding, reflective value-judgment, and interaction-action specify the analytical dimensions; and textbook content, reflection prompts, and communicative tasks serve as design-level indicators. Although the three-dimensional framework is constructed for the purposes of the present textbook analysis, it is not an ad hoc model. Its formal logic derives from Klieme’s competence-model approach, while its content dimensions are grounded in intercultural competence research, ESD scholarship, social cognitive theory, experiential learning theory, and affordance theory. Following Klieme, competence is understood not only as cognitive ability but also as motivational, volitional, and social dispositions that enable responsible action in changing situations. This links competence to action, responsibility, and pedagogical operationalization.

On this Klieme-informed basis, German-language scholarship helps specify the intercultural content of the framework. Bolten understands intercultural action competence as a multidimensional capacity involving individual, social, professional, and strategic dimensions, while process-oriented accounts stress negotiation, appropriateness, and interactional adjustment (Bolten, 2001, 2016, 2018; Knapp, 1995). This scholarship supports treating intercultural competence not as the accumulation of cultural facts but as a dynamic and teachable capacity involving cognition, reflection, and action. It also makes it possible to reinterpret intercultural competence as a set of learnable behaviors rather than only as a static competence structure.

ESD reshapes intercultural competence by foregrounding sustainability literacy, responsibility, justice, and the relation between language learning and ecological, social, and economic issues (Ge et al., 2023; Schreiber & Siege, 2016; Wals & Kieft, 2010). Learning behavior theory further clarifies how these aims may be pedagogically mediated. Social cognitive theory highlights the reciprocal relation among personal factors, behavior, and environment, including self-regulatory and self-reflective processes (Bandura, 1986). Experiential learning theory similarly stresses the movement from experience to reflection, conceptualization, and active experimentation (Kolb, 1984). In this perspective, textbooks can be treated as pedagogical environments that provide design-level affordances for observing, comparing, reflecting, evaluating, communicating, and acting.

The article does not claim that “sustainable intercultural competence” is a standardized or widely established competence construct. The term is used only as a working analytical shorthand for the sustainability-oriented development of intercultural competence. In Klieme’s competence-model logic, broad educational aims do not automatically constitute a new competence; they must be translated into domain-specific dimensions and operational indicators. Accordingly, this study does not validate a new psychometric construct. It examines how intercultural competence may be reoriented under sustainability-related aims and how this reorientation is made visible in textbook design. The framework therefore operationalizes sustainability-oriented intercultural competence development as textbook-embedded learning-behavioral affordances, not as evidence of learners’ actual behavior (Aronin & Singleton, 2012; Nguyen, 2025). Thus, the framework is primarily an analytical tool for textbook analysis rather than a validated pedagogical model or a psychometric competence construct. It builds on established intercultural competence frameworks by retaining the concern with cultural understanding, reflection, and interaction but extends them by asking how sustainability-related responsibility, future orientation, and action are pedagogically embedded in textbook design.

**Figure 1:**
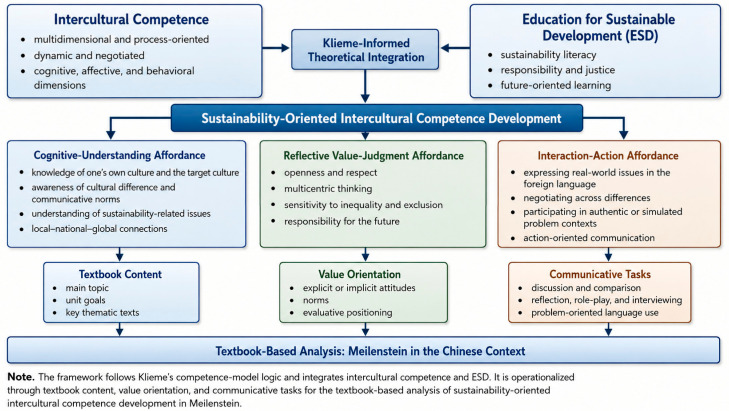
Klieme-informed framework of learning-behavioral affordances for sustainability-oriented intercultural competence development.

As shown in Figure 1, the framework follows Klieme’s competence-model logic and integrates intercultural competence research, ESD scholarship, and learning-behavioral affordance theory (Bolten, 2018; Byram, 1997; Ge et al., 2023; Klieme et al., 2003; Nguyen, 2025). It specifies three affordance dimensions: cognitive-understanding, reflective value-judgment, and interaction-action. Cognitive-understanding affordances concern opportunities to notice, compare, explain, and interpret cultural, social, and sustainability-related meanings. Reflective value-judgment affordances concern opportunities to examine assumptions, articulate values, evaluate responsibility, and reflect on inequality, exclusion, environmental degradation, and future-oriented coexistence. Interaction-action affordances concern opportunities to express real-world issues in German, negotiate across differences, mediate perspectives, and participate in communicative or problem-oriented tasks. The three dimensions are operationalized through textbook content, reflection prompts, value orientation, and communicative tasks. They are treated as design-level affordances rather than direct evidence of learners’ actual behavior.

### 1.3. Previous Research and Research Gap

Research in foreign-language education has long established that language learning cannot be separated from culture. Within this tradition, intercultural competence has become one of the most influential concepts in language pedagogy. Earlier work challenged the view of culture as supplementary background knowledge and instead treated it as constitutive of language learning itself (Byram, 1997; Kramsch, 1993). Subsequent work criticized methodological nationalism and the static coupling of language, nation, and culture, calling instead for transnational, culture-reflexive, and discourse-oriented approaches to cultural learning (Feike, 2019; Monteiro, 2007; Risager, 2005). More recent studies have emphasized cultural and intercultural awareness as flexible and context-sensitive, positioning learners as participants in complex communicative and social worlds rather than as mere recipients of cultural facts (Baker, 2015, 2022). This scholarship has established intercultural competence as a core educational objective, but it has paid less attention to how it may be reformulated in relation to sustainable development and learning behavior (Baker, 2022; Bolten, 2018; Byram, 1997; Byram & Wagner, 2018). Recent behavioral sciences research supports such a behavior-oriented extension by treating intercultural effectiveness as the behavioral aspect of ICC and by emphasizing communicative adaptation, verbal and non-verbal skills, and effective interaction across cultural differences (Beltrán-Véliz et al., 2024).

A second strand of research concerns ESD and sustainability literacy in language education. The main shift here has been a gradual shift from treating sustainability as a thematic supplement to viewing it as a framework with implications for educational aims, classroom content, and learner participation. Studies in this area emphasize that sustainability is not confined to environmental protection but involves ecological, social, economic, and ethical interdependence (UNESCO, 2018; Schreiber & Siege, 2016; Svalfors, 2017; Vare & Scott, 2007; Wals & Kieft, 2010). In language education, this means that foreign-language learning is increasingly seen as directly relevant to sustainable development because global problems are necessarily mediated through language, discourse, and intercultural communication. Zygmunt, for example, explicitly frames language education as part of sustainable development, arguing that participation in sustainability-related communication requires sociolinguistic competence, tolerance, and awareness beyond the local scale (Zygmunt, 2016). More recent work has extended this perspective by conceptualizing language education for sustainability as transformative, reflective, and action-oriented, while systematic reviews emphasize key competences for ESD-oriented curriculum design (D. Müller, 2022; Vare & Scott, 2007). This emphasis is consistent with recent work on self-sustained language learning, which links sustainability-oriented education with learners’ active, persistent, and self-initiated pursuit of language development (Yang et al., 2024). German-language debates warn that the ambitious programmatics of Bildung für nachhaltige Entwicklung (BNE) do not automatically translate into educational practice (Budde & Blasse, 2023). In German as a foreign language, similar arguments have been made for integrating ESD into university teaching and for using language classrooms as spaces in which learners reflect on the consequences of their own actions, encounter multiple perspectives, and develop more globally oriented forms of understanding (Kapranov, 2021; Y. Li & Hua, 2025; D. Müller, 2022). Corpus-based work on SDG-related discourse further suggests that sustainability themes can be traced productively through systematic language analysis, even beyond explicitly pedagogical corpora (Pinto, 2021).

A third body of the literature is directly concerned with textbooks. Recent textbook research has moved beyond viewing textbooks as neutral carriers of linguistic input and instead treats them as sites of representation, interaction, and learning (Canale, 2021). This perspective is particularly relevant for the present study because it opens textbook analysis to questions of worldview, value orientation, and pedagogical design. Canale argues that textbooks should be approached not only as pedagogical tools but also as cultural and ideological artefacts that organize forms of interaction and learning (Canale, 2021). Similarly, research on English textbooks in China has proposed frameworks for examining how global competence is represented through content and task design, thereby showing that textbooks can be analyzed in relation to broader educational goals rather than language learning alone (Ping & Wang, 2023). Within ESD-related textbook studies, recent work has shown that language materials may contribute to sustainability awareness but that the distribution of sustainability themes is often uneven and that social justice, value formation, and action-related competences remain underdeveloped (Sachdev & Vibulphol, 2025). The existing literature thus confirms that textbooks matter for sustainability-oriented education, but it also indicates that the mere inclusion of themes is insufficient. What matters is how knowledge, values, and communicative practices are pedagogically organized as affordances for learning action (Aronin & Singleton, 2012; Canale, 2021; Nguyen, 2025; Ping & Wang, 2023; Sachdev & Vibulphol, 2025). From this perspective, textbook texts, questions, tasks, and projects can be analyzed as designed opportunities for learners to interpret, evaluate, interact, and act (Aronin & Singleton, 2012; Nguyen, 2025).

Research specifically focused on German-language teaching materials in China remains comparatively limited. Existing Chinese studies on German textbooks have mainly concentrated on cultural representation, especially the distribution of German-speaking cultures and Chinese culture in widely used teaching materials (Ge, 2022). At the same time, recent German-language scholarship on cultural learning in German as a foreign or second language (DaF/DaZ) has diagnosed an unfinished paradigm shift from traditional Landeskunde toward culture-reflexive learning, suggesting that textbook analysis must attend not only to content but also to discourse participation and meaning construction (Feike, 2019; Monteiro, 2007). Research on sustainable foreign-language education in China has begun to draw attention to multilingual development, global issues, and the cultivation of sustainable citizens, but it has not yet yielded a textbook-based model for analyzing the sustainable development of intercultural competence in German-language education (Ge et al., 2023; Y. Li & Hua, 2025). In other words, the available literature tends either to discuss interculturality without sustainability or sustainability without a sufficiently fine-grained intercultural and learning-behavioral framework for textbook analysis.

The foregoing review points to three connected but distinct gaps. First, existing intercultural competence research has not sufficiently examined how intercultural learning can be reoriented toward sustainability-related responsibility and future-oriented action. Second, ESD-oriented language education research has paid limited attention to the textbook-level learning behaviors through which sustainability aims are pedagogically scaffolded. Third, research on German textbooks used in China has focused mainly on cultural representation, while the joint organization of textbook content, value orientation, and communicative tasks as learning-behavioral affordances remains underexplored.

### 1.4. Present Study and Research Questions

To address this gap, this article focuses on *Meilenstein*, a recently published four-volume textbook series designed for university-level German-major instruction in China. Its complete progression across four volumes, national publishing platform, and coverage of everyday, social, intercultural, sustainability-related, and reflective themes make it a useful case for examining how German-language teaching materials shape intercultural learning in the Chinese context. The central argument is that intercultural competence in foreign-language education needs to be reconsidered in relation to ESD and learning behavior and that this reconsideration requires a Klieme-informed analytical framework capable of translating broad educational aims into domain-related dimensions and operational indicators (Bolten, 2018; Ge et al., 2023; Klieme et al., 2003; Schreiber & Siege, 2016; Vare & Scott, 2007; Wals & Kieft, 2010). The article therefore develops a three-dimensional framework of learning-behavioral affordances for sustainability-oriented intercultural competence development and uses it to examine how *Meilenstein* organizes design-level opportunities for cognitive understanding, reflective value judgment, and interaction-oriented action. Specifically, the study asks:

(1) What types of textbook-embedded learning-behavioral affordances for sustainability-oriented intercultural competence development are provided by *Meilenstein*?
(2) How are cognitive-understanding, reflective value-judgment, and interaction-action affordances distributed across units, volumes, and pedagogical components?
(3) What are the major strengths and limitations of *Meilenstein* in scaffolding sustainability-oriented intercultural learning behaviors in the Chinese context?

## 2. Materials and Methods

### 2.1. Research Design

The study uses a qualitatively driven mixed-method design, combining directed qualitative content analysis with descriptive frequency aggregation. This design was chosen because the research does not aim to test causal relationships or measure learners’ actual classroom behavior but to interpret textbook-embedded affordances while using descriptive numerical aggregation to clarify distributional patterns. Following an affordance-oriented view of language education, the study analyzes whether textbook texts, prompts, and tasks create opportunities for cognitive understanding, reflective value judgment, and interaction-oriented action (Aronin & Singleton, 2012; Nguyen, 2025). Content analysis is suitable for this purpose because it allows rule-guided interpretation of textual and pedagogical material while remaining sensitive to context and category formation (Krippendorff, 2018; Mayring, 2000). Krippendorff defines content analysis as a research technique for making replicable and valid inferences from texts to the contexts of their use (Krippendorff, 2018), while Mayring emphasizes its stepwise and category-based character, especially in studies that combine theoretical guidance with qualitative interpretation (Mayring, 2000).

This study follows a directed or theory-guided form of qualitative content analysis. As Hsieh and Shannon note, directed content analysis begins with existing theory or prior research as guidance for initial coding categories (Hsieh & Shannon, 2005). In the present study, these categories are provided by the three-dimensional framework of textbook-embedded learning-behavioral affordances: cognitive-understanding affordance, reflective value-judgment affordance, and interaction-action affordance. Their application, however, remains interpretive rather than mechanical because the coding process requires contextual judgment about how textbook content, value orientation, and communicative tasks contribute to the three dimensions. The study therefore combines deductive category application with iterative reading and revision rather than relying on a purely inductive procedure (Hsieh & Shannon, 2005; Mayring, 2000).

### 2.2. Materials

The data for this study consist of the four volumes of *Meilenstein* (J. Li, 2023; Zhan, 2024; Y. Li, 2025; Kong, 2025). All four volumes are first-edition textbooks published by Foreign Language Teaching and Research Press under the general editorship of Wenjian Jia. The individual volume editors are Jing Li for Volume 1, Xia Zhan for Volume 2, Yuan Li for Volume 3, and Deming Kong for Volume 4. The four volumes were published in 2023, 2024, and 2025, respectively. The corpus includes 37 units in total: nine units in Volume 1, 12 in Volume 2, and eight each in Volumes 3 and 4. The complete series was analyzed in order to identify broader pedagogical patterns across volumes and thematic progression. *Meilenstein* was selected for three reasons: it is a complete four-volume series designed for university-level German-major education in China; it covers a broad thematic progression from everyday communication to social, cultural, sustainability-related, and reflective topics; and it allows the analysis of how learning-behavioral affordances develop across textbook levels rather than within a single isolated volume.

Recent textbook research no longer treats textbooks as neutral carriers of linguistic input but as sites of representation, interaction, and learning (Canale, 2021). The present analysis follows this view. It therefore considers not only thematic content but also the ways in which units organize intercultural knowledge, value positioning, communicative engagement, and the learning-behavioral affordances invited by these pedagogical components. Ping and Wang’s framework-based approach likewise shows that textbook material can be examined in relation to broader educational aims and non-linguistic learning dimensions (Ping & Wang, 2023). The corpus was therefore analyzed at the level of the complete unit, while also taking into account embedded components such as learning objectives, main texts, dialogues, discussion prompts, and communicative tasks.

### 2.3. Analytical Framework and Units of Analysis

The analysis is guided by the Klieme-informed three-dimensional framework of textbook-embedded learning-behavioral affordances developed in the previous section. The first dimension, cognitive-understanding affordance (CU), concerns the extent to which textbook materials invite learners to notice, identify, compare, explain, and interpret cultural, social, and sustainability-related issues. The second dimension, reflective value-judgment affordance (VJ), refers to the extent to which the material encourages learners to articulate values, examine assumptions, evaluate responsibility, and respond to issues such as inequality, exclusion, environmental degradation, and future-oriented coexistence. The third dimension, interaction-action affordance (IA), concerns the extent to which the textbook enables learners to express real-world issues in the target language, negotiate across differences, mediate perspectives, and participate in communicative or task-based activities involving discussion, comparison, roleplay, proposal-making, or problem-oriented engagement. In line with Klieme’s competence-model logic, these three dimensions function as operationalized analytical dimensions rather than as direct measures of learners’ actual competence.

The items grouped under the three dimensions do not come from a single existing instrument. They were put together from the two strands of the literature discussed above. In the cognitive-understanding dimension, cultural knowledge, communicative norms, and awareness of cultural difference are drawn mainly from intercultural competence research (Baker, 2015, 2022; Bolten, 2016, 2018; Byram, 1997; Byram & Wagner, 2018; Feike, 2019; Knapp, 1995; Kramsch, 1993; Liu, 2003; Monteiro, 2007; Pan, 2008; Risager, 2005; S. Müller & Gelbrich, 2004). The attention to sustainability-related issues in local, national, and global contexts comes more directly from the ESD and sustainability literacy literature (Budde & Blasse, 2023; Ge et al., 2023; González-Salamanca et al., 2020; UNESCO, 2018; Kapranov, 2021; Y. Li & Hua, 2025; D. Müller, 2022; Schreiber & Siege, 2016; Stibbe, 2008; Svalfors, 2017; Vare & Scott, 2007; Wals & Kieft, 2010; Zygmunt, 2016). In the value-judgment dimension, openness, respect, and multicentric thinking follow mainly from intercultural competence scholarship (Baker, 2015, 2022; Borghetti, 2017; Feike, 2019; Knapp, 1995; Liu, 2003; S. Müller & Gelbrich, 2004; Pan, 2008), whereas sensitivity to inequality, exclusion, environmental degradation, and future responsibility reflects the stronger influence of ESD-oriented work (Budde & Blasse, 2023; Ge et al., 2023; González-Salamanca et al., 2020; UNESCO, 2018; Kapranov, 2021; Y. Li & Hua, 2025; D. Müller, 2022; Schreiber & Siege, 2016; Stibbe, 2008; Svalfors, 2017; Vare & Scott, 2007; Wals & Kieft, 2010; Zygmunt, 2016). In the interaction-action dimension, expression, negotiation, mediation, and participation in communicative problem contexts are rooted mainly in process-oriented accounts of intercultural competence (Bolten, 2001, 2016; Knapp, 1995; S. Müller & Gelbrich, 2004). Its action-oriented and future-oriented emphasis, however, is shaped more clearly by sustainability-oriented language education (Kapranov, 2021; Y. Li & Hua, 2025; D. Müller, 2022; Zygmunt, 2016). In the present study, these items function as Klieme-informed operational indicators of designed learning-behavioral affordances rather than as a psychometric scale for measuring learners’ actual competence.

In operational terms, the primary unit of analysis in this study is the unit/lesson. This choice reflects the fact that in a textbook series such as *Meilenstein,* the unit represents the most coherent pedagogical cluster in which theme, objectives, texts, and tasks are systematically combined. At the same time, unit-level coding was informed by three embedded analytical entry points: (1) textbook content, including the main topic, unit goals, and key thematic texts; (2) value orientation, including explicit or implicit attitudes, norms, and evaluative positioning; and (3) communicative tasks, including prompts for discussion, comparison, reflection, roleplay, interviewing, or problem-oriented language use. This combination of a unit-level primary coding structure with text- and task-sensitive reading is consistent with recent approaches that analyze textbooks as articulated configurations of content, interaction, and learning (Canale, 2021; Ping & Wang, 2023). To increase coding transparency and consistency, the three dimensions were further operationalized through a set of guiding questions and indicators, as summarized in Table 1.

To make the coding procedure more transparent, Table 1 summarizes the operational indicators used to identify the three dimensions in the corpus. The table specifies the main coding question for each dimension and the types of indicators treated as primary evidence during coding. Because the dimensions were not treated as mutually exclusive categories, a single unit or embedded item could support more than one dimension; the coding therefore assessed the relative strength of each dimension rather than forcing exclusive classification.

### 2.4. Coding Procedure

The coding process unfolded in four stages. First, all four volumes were read in full in order to establish familiarity with the overall structure, progression, and thematic distribution of the series. Preliminary notes focused on recurring themes, intercultural content, sustainability-related issues, and typical task formats. The second stage consisted of a unit scan covering titles, learning objectives, major texts, and visible task structures. This made it possible to identify the general pedagogical orientation of each unit and to note whether the three learning-behavioral affordance dimensions were absent, implicit, or explicitly foregrounded.

In the third stage, each unit was coded on a three-point scale: 0 = no clear behavioral affordance, 1 = limited or implicit behavioral affordance, and 2 = explicit and sustained behavioral affordance. The 0–2 scale used in this study follows the principles of ordinal coding commonly applied in content analysis and curriculum evaluation. Similar to the variable-based analytical scheme developed by Weiss and Barth (2020) for analyzing sustainability curricula across higher education institutions, each affordance dimension (CU, VJ, IA) was operationalized with clear criteria for absent (0), limited/implicit (1), and explicit/sustained (2) occurrences. Coding decisions were documented systematically, and independent checking by the corresponding author ensured consistency across units and volumes. This approach supports transparency, replicability, and descriptive aggregation of textbook affordances, without implying psychometric measurement of student competencies.

To make the scoring procedure more transparent, Table 2 provides a concise illustration of how scores of 0, 1, and 2 were differentiated across the three affordance dimensions. The table does not reproduce extensive textbook material; instead, it summarizes the coding logic through representative paraphrased examples. A fuller set of purposively selected sample coding decisions is provided in Table S6 in the Supplementary Materials; these examples cover different volumes, thematic clusters, score levels, and both strong and boundary cases.

This score differentiation was used consistently during unit-level coding and was checked against the operational indicators in Table 1. Because the study is theory-guided, the coding did not begin with open-ended categories derived solely from the material. It started from the three affordance dimensions as prior categories, while still relying on repeated reading and revision in the interpretation of each unit (Hsieh & Shannon, 2005; Mayring, 2000).

The initial coding of all 37 units was conducted by the first author. To strengthen interpretive reliability, the corresponding author independently checked the coding table and reviewed a subset of units representing different volumes, thematic clusters, and score patterns. Particular attention was given to borderline cases, especially where the distinction between CU and IA was not immediately clear or where VJ was present only implicitly. Disagreements and ambiguous cases were discussed until consensus was reached. The final scores therefore reflect consensus-based coding rather than unverified single-reader interpretation.

The final stage consisted of short analytical notes for each unit. These notes recorded the reasons for the assigned scores and identified representative examples. They helped move the analysis from individual coding decisions to broader interpretive patterns across the series. At a later point, the units were also grouped thematically in order to identify recurring clusters such as everyday life, family and relationships, customs and identity, media and digitalization, sustainability and environment, and language/ literature/knowledge. The thematic clusters were formed inductively after unit-level coding by comparing unit titles, stated learning objectives, main texts, and dominant communicative tasks. Units were grouped according to their primary thematic orientation rather than according to the three affordance scores. When a unit could fit more than one cluster, the dominant topic and main pedagogical focus were used as the deciding criteria. This thematic clustering did not replace the primary coding system. It served as a second-order analytical step for synthesizing the findings across all 37 units. The procedure follows Mayring’s emphasis on rule-guided category work and on the transparency of code revision and aggregation (Elo & Kyngäs, 2008; Mayring, 2000).

### 2.5. Validity and Reliability

Given the qualitatively driven and interpretive orientation of the study, validity and reliability were addressed through transparent indicators, consistent use of the coding framework in Table 1, score differentiation criteria in Table 2, independent checking by the corresponding author, repeated reading of borderline cases, and consensus discussion rather than through large-scale statistical testing. The coding procedure relied on explicit operational definitions of the three analytical dimensions and on a fixed scoring scale (0–2). The indicators in Table 1 and the score differentiation criteria in Table 2 made it easier to distinguish between materials that mainly afforded CU behaviors, those that primarily scaffolded reflective VJ behaviors, and those that more clearly invited IA behaviors. Ambiguous units were reread and checked again, especially where value orientation remained implicit or where the boundary between CU and IA was not immediately clear. Coding decisions were also documented in unit-level, focal-unit, and sample-coding tables so that the analytical procedure remained traceable. In particular, Table S6 provides representative coding decisions, paraphrased evidence, and coding rationales to clarify how the 0–2 scoring scale was applied in practice. Because the study examines textbook design rather than learner uptake, it does not claim that the identified affordances were necessarily perceived or enacted by learners (Nguyen, 2025).

This approach is consistent with the qualitative content analysis literature, which stresses rule-guided interpretation, category-based analysis, and the need to make analytical decisions intersubjectively comprehensible (Krippendorff, 2018; Mayring, 2000). Mayring, in particular, argues that reliability and validity in qualitative content analysis depend not only on formal quantification but also on clearly defined procedures, category revision, and feedback loops (Mayring, 2000). Hsieh and Shannon make a similar point for theory-guided content analysis, where pre-established categories can shape interpretation if they are not applied critically and transparently (Hsieh & Shannon, 2005). In the present study, this risk was addressed by repeatedly checking whether the coding was genuinely supported by unit content and task design rather than imposed on the material by the analytical model.

### 2.6. Data Analysis

The analysis combines qualitative interpretation with descriptive frequency aggregation within a qualitatively driven mixed-method design. The qualitative component is primary: it focuses on how individual units provide learning-behavioral affordances through thematic content, value orientation, reflection prompts, and communicative tasks. The descriptive quantitative component is secondary: it consists of aggregating the 0–2 scores across units, volumes, and thematic clusters in order to identify broader distributional patterns. This mixed but modest use of frequency counts follows the logic of qualitative content analysis as described by Elo and Kyngäs, who note that qualitative category work can also support broader conceptual description and comparison (Elo & Kyngäs, 2008).

More specifically, the coded scores were used in three ways. First, they were aggregated by volume in order to identify how the three dimensions are distributed across the four volumes of *Meilenstein*. Second, they were aggregated by thematic unit cluster in order to reveal broader pedagogical patterns beyond individual lessons. Third, the full unit-by-unit coding table was retained in Supplementary Materials so that the summary patterns in the main text would remain analytically transparent. In this way, the study does not reduce textbook analysis to numerical comparison alone. Rather, frequency counts are used to support and clarify interpretive findings about the strengths, limitations, and internal distribution of sustainability-oriented intercultural competence development in the textbook series (Canale, 2021; Elo & Kyngäs, 2008; Ping & Wang, 2023). Descriptive aggregation, tables, and figures were prepared using Microsoft Excel for Microsoft 365. Reference management was conducted using Mendeley Cite. No custom computer code was used in the analysis.

## 3. Results

### 3.1. Overall Distribution of Learning-Behavioral Affordances Across the Four Volumes

**Figure 2:**
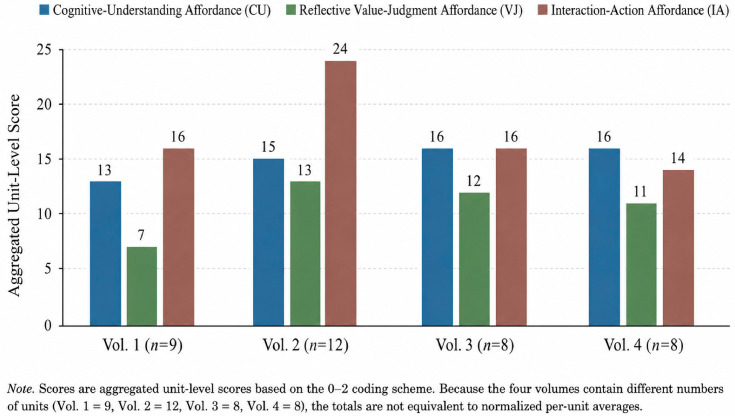
Distribution of the three learning-behavioral affordance dimensions across the four volumes of *Meilenstein*.

Figure 2 presents the volume-level distribution of the three learning-behavioral affordance dimensions, and Table S2 provides the corresponding summary. Across all 37 units, IA received the highest aggregated score (70), followed by CU (60), whereas VJ was substantially lower (43). This overall distribution supports the central finding that *Meilenstein* more consistently scaffolds communicative participation and cognitive understanding than explicit reflective value judgment. Because the four volumes contain different numbers of units, the volume-level totals in Figure 2 should be interpreted as aggregated unit-level scores rather than normalized per-unit averages. The results show an uneven but clear distribution across the series. In Volume 1, IA is the strongest dimension, followed by CU, while VJ remains clearly weaker. In Volume 2, IA again reaches the highest total. This should be read in light of the fact that Volume 2 contains 12 units, whereas Volumes 1, 3, and 4 contain nine, eight, and eight units, respectively. Its stronger interaction-action profile therefore reflects not only communicative orientation but also the larger number of units. In Volume 3, CU and IA are equally strong, whereas VJ remains lower. In Volume 4, CU is the strongest dimension, IA remains relatively high, and VJ is again the weakest of the three. Overall, *Meilenstein* provides stronger affordances for cognitive-understanding and interaction-action behaviors than for reflective value-judgment behaviors.

The internal profile of the series also changes across the four volumes. Volume 1 is largely shaped by interaction-oriented everyday intercultural learning, with relatively strong support for communicative practice in units dealing with campus life, transport, shopping, health, food, and festivals. Volume 2 retains a strong communicative orientation, but it moves more clearly toward social and relational issues, especially in units on family, customs, media, intergenerational internet use, and smartphone dependence. Volume 3 is the most balanced volume. Figure 2 shows equal strength in cognitive-understanding and interaction-action and a comparatively stronger value-judgment dimension than in the earlier volumes. Volume 4 shifts toward a more abstract and reflective profile. Its units address youth values, responsibility, identity, exchange, art, literature, and knowledge. For that reason, it remains strong in cognitive-understanding, while value-judgment and interaction-action are distributed less evenly across the volume.

Two tendencies stand out in Figure 2. First, interaction-action is most prominent in the earlier part of the series, especially in Volumes 1 and 2. This confirms that the lower levels of the textbook prioritize communicative participation in everyday and social situations. Second, value-judgment remains lower than the other two dimensions in all four volumes. Even where value-related themes become more visible in Volumes 3 and 4, this dimension does not overtake either cognitive-understanding or interaction-action. The series is therefore more consistent in promoting intercultural understanding and communicative engagement than in fostering explicit evaluative reflection.

### 3.2. Thematic Patterns of Learning-Behavioral Affordances Across Unit Clusters

**Figure 3:**
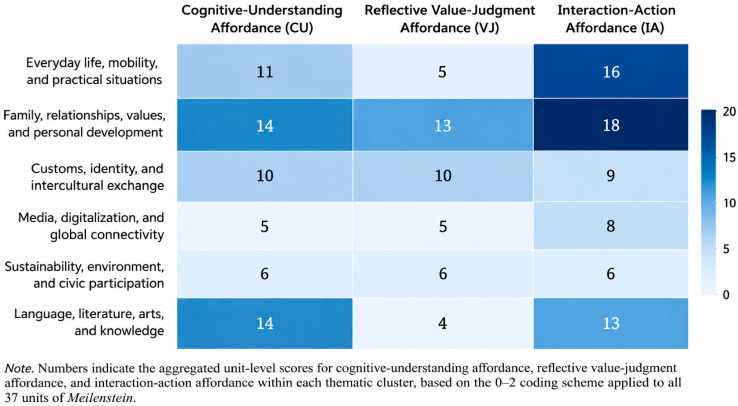
Distribution of learning-behavioral affordances across thematic unit clusters in *Meilenstein*.

To answer the second research question in greater detail, the units were grouped thematically according to their dominant topic and pedagogical focus in order to identify broader patterns across the series. The clusters reported in Figure 3 were formed independently of the CU/VJ/IA scores so that the thematic grouping would not simply reproduce the coding results. Figure 3 shows substantial variation across thematic clusters, and Table S3 summarizes the corresponding aggregated unit-level scores. At the thematic-cluster level, the highest total score appears in “Family, relationships, values, and personal development” (45), followed by “Everyday life, mobility, and practical situations” (32), “Language, literature, arts, and knowledge” (31), and “Customs, identity, and intercultural exchange” (29). The “Media, digitalization, and global connectivity” and “Sustainability, environment, and civic participation” clusters each have a lower total score (18), but the latter is internally balanced across CU, VJ, and IA (6/6/6). The cluster “Family, relationships, values, and personal development” is the strongest overall, with comparatively high values in all three dimensions. Units in this cluster are especially effective in linking lived experience, social values, and communicative engagement. They encourage learners to talk about themselves and others, but they also invite reflection on values, life choices, and social relations.

A second strong cluster is “Customs, identity, and intercultural exchange”. Here, the pattern is relatively balanced between CU and VJ, while IA is slightly lower. Such units are particularly rich in intercultural comparison and make implicit value orientations visible through topics such as gift-giving, politeness, festivals, customs, identity, and exchange. The textbook moves beyond factual cultural presentation and encourages learners to compare norms, expectations, and symbolic meanings across cultural contexts. The slightly lower interaction-action value points to a modest relative emphasis on cultural understanding and evaluative comparison over extended action-oriented participation.

The cluster “Everyday life, mobility, and practical situations” shows a different pattern. Here, IA is clearly dominant, while VJ remains comparatively weak. This is hardly surprising, since many units in this group focus on asking for directions, making appointments, shopping, or handling other practical situations. Such units are strong in communicative functionality and everyday intercultural knowledge, but they rarely invite sustained reflection on responsibility, inequality, or longer-term social consequences. A similar tendency appears in “Media, digitalization, and global connectivity”, where discussion and social relevance are present, but all three dimensions remain only moderately developed.

The cluster “Sustainability, environment, and civic participation” matters especially for the present study because it contains the most explicit concentration of sustainability-related themes in the series. Although the number of units is small, the distribution across the three dimensions is relatively balanced. Units on climate change, sustainability, and engagement connect intercultural learning more directly with environmental and civic concerns and show more clearly than most other parts of the textbook how sustainability-oriented intercultural learning is built into the material. By contrast, “Language, literature, arts, and knowledge” is strong in CU and relatively strong in IA but noticeably weaker in VJ. The more abstract and disciplinary units in the later volumes therefore support analytical understanding and communicative interpretation more strongly than explicit value reflection.

### 3.3. Strengths and Limitations of Learning-Behavioral Affordances at the Unit Level

The full unit-level results clarify how these broader patterns are realized in specific lessons. Table S1 presents the complete coding results for all 37 units. At the unit level, *Meilenstein* shows both strong and weak affordance patterns. Units dealing with family, customs, climate change, sustainability, civic engagement, youth values, social responsibility, and identity provide the strongest and most balanced affordances for sustainability-oriented intercultural learning. By contrast, units centered on practical communication, job application, or disciplinary knowledge tend to privilege CU or IA while offering fewer opportunities for reflective VJ. This confirms the broader pattern visible in Figure 2 and Figure 3: the series provides a relatively strong basis for intercultural understanding and communicative action, but value-oriented reflection remains more selective and uneven. The next section therefore examines selected focal units to show how texts, tasks, and reflection prompts organize these affordance patterns internally.

### 3.4. Fine-Grained Analysis of Selected Focal Units

Tables S4 and S5 provide a fine-grained analysis of eight focal units. These units were selected because they contain the clearest articulation of intercultural or sustainability-related content across the four volumes. Across the focal units, theme texts most consistently afford CU behaviors, communicative tasks are the main carriers of IA affordances, and reflection prompts are the main site of reflective VJ affordances. This internal division of pedagogical labor helps explain why value judgment is present across the series but remains less evenly scaffolded than the other two types of learning behavior.

Five examples illustrate this pattern. In Volume 2, Lektion 11, “Nie zu alt fürs Internet!”, digital communication provides opportunities for learners to compare communicative norms, interpret online interaction, and discuss responsible media use. The unit is relatively strong in CU and IA, but VJ depends mainly on whether discussion tasks are extended into explicit reflection on responsibility, inclusion, and digital ethics.

In Volume 3, Lektion 5, “Klimawandel und Umweltschutz” (pp. 153–172), sustainability-related content is most explicitly integrated with communicative action and value reflection. The unit introduces climate change, carbon footprints, extreme weather, adaptation, and environmental responsibility as transnational issues. It affords CU through conceptual and factual input, IA through discussion and problem-solving tasks, and VJ through prompts on personal environmental goals, responsibility, and consequences for future generations.

Volume 3, Lektion 6, “Rund um die Nachhaltigkeit,” extends this orientation by connecting sustainability with everyday practices and lifestyle choices. Its value lies in moving sustainability from abstract knowledge to daily decision-making. The unit provides CU through sustainability-related concepts, IA through discussion and proposal-making, and VJ through reflection on consumption, responsibility, and future-oriented action.

In Volume 4, Lektion 2, “Individuum und soziale Verantwortung” (pp. 31–50), the focus shifts from environmental responsibility to personal and social responsibility. The unit connects personal development, moral action, reliability, leadership, and social engagement. Its strongest affordances lie in CU and VJ because learners are invited not only to understand responsibility-related concepts but also to reflect on what responsible action means in personal and social life. IA is present but less concrete than in the climate-change unit.

Volume 4, Lektion 3, “Heimat und Identität” (pp. 71–90), provides another reflective pattern. It links belonging, cultural affiliation, migration-related experience, and identity. The unit is strong in CU and VJ because it invites learners to interpret complex social meanings and reflect on self-positioning in culturally diverse contexts. However, IA remains more reflective than solution-oriented. Taken together, these examples show that the most balanced units are those in which texts, tasks, and prompts connect knowledge, judgment, and communicative action within the same pedagogical sequence.

## 4. Discussion

### 4.1. From Cultural Input to Cognitive Learning Behavior

*Meilenstein* provides learning-behavioral affordances for sustainability-oriented intercultural competence development in a differentiated rather than uniform way. As Figure 2 shows, CU and IA affordances are consistently stronger than reflective VJ affordances across the four volumes. The series is therefore more effective in turning cultural and sustainability-related input into opportunities for understanding and communicative participation than in sustaining explicit reflection on values, responsibility, inequality, or future-oriented coexistence. From a learning-behavioral perspective, this means that the textbook places greater emphasis on knowing and interacting than on judging and positioning (Bandura, 1986; Nguyen, 2025). The imbalance is most visible in the earlier volumes, where everyday situations create many opportunities for communicative practice but relatively few for explicit ethical reflection.

This pattern suggests that thematic progression alone is insufficient to generate reflective value judgment. Even when later volumes include more abstract social and cultural themes, explicit evaluative scaffolding depends on whether tasks require learners to justify positions, compare values, or connect reflection with possible action. The continued relative weakness of VJ therefore indicates not simply a thematic gap but a task-design gap: value-related topics become behaviorally meaningful only when they are transformed into structured opportunities for reflection, evaluation, and positioning.

### 4.2. Reflective Value-Judgment as a Weak Behavioral Link

The weaker VJ dimension is theoretically significant. Textbooks are not neutral containers of linguistic input but configurations of representation, interaction, and learning (Canale, 2021). In *Meilenstein*, the pedagogical effect depends not simply on whether intercultural or sustainability-related topics are present but on how content, value orientation, and tasks are combined. This point is consistent with work showing that broader educational aims, including global competence and sustainability-related learning, can be traced through textbook design rather than only through overt thematic content (Ping & Wang, 2023; Sachdev & Vibulphol, 2025).

From a learning-behavioral perspective, value judgment is not merely the presence of ethical themes. It refers to whether learners are invited to examine assumptions, articulate values, justify positions, and connect reflection with action. Bandura’s account of self-regulation and self-reflection explains why this matters: learners need opportunities to monitor, evaluate, and revise their own thinking. Kolb’s experiential learning theory similarly treats reflection as a link between experience and active experimentation (Kolb, 1984). The present findings therefore extend Chinese research on German textbooks, which has mainly focused on cultural representation and cultural balance, by showing that textbook units should also be examined in terms of whether they scaffold judgment, self-positioning, and responsibility (Ge, 2022).

### 4.3. Interaction-Action and the Transition from Knowing to Doing

From a theoretical perspective, the findings show that sustainability-oriented intercultural competence development should be understood not as a fixed content-based construct but as a behavioral sequence linking knowing, judging, and acting. The three-dimensional framework retains its explanatory value because it distinguishes among dimensions that would remain blurred in a general analysis of “culture” or “interculturality”. The interaction-action dimension is central to this sequence because it moves intercultural learning beyond understanding toward communicative participation. Recent research on intercultural effectiveness defines the behavioral aspect of ICC in terms of verbal and non-verbal communicative skills, behavioral adaptation, and effective interaction across cultural differences (Beltrán-Véliz et al., 2024). This supports the interpretation of textbook discussion tasks, role plays, mediation tasks, and proposal-making activities as design-level affordances for intercultural action.

The findings also show that ESD does not replace intercultural competence but reorients its analytical and pedagogical interpretation. In the present study, sustainability becomes visible not only in units explicitly dealing with climate change or sustainability but also in the ways units frame happiness, family, digital media, responsibility, or identity. Sustainability in language education should therefore not be reduced to environmental content alone. It is more usefully understood in relation to social, cultural, ethical, and civic dimensions. A sustainability-oriented intercultural analysis can thus capture both explicit and implicit forms of pedagogical orientation. In the Chinese higher education context, intercultural communication competence has also been shown to predict students’ willingness to form intercultural friendships with international students, which suggests that classroom-based interaction-action tasks may have relevance beyond textbook practice (Tang & Zhang, 2023).

### 4.4. Implications for Behaviorally Informed Textbook Design

The findings are relevant not only for textbook analysis but also for classroom practice and future textbook design. These implications concern textbook design and teacher mediation rather than demonstrated learner outcomes because the present study analyzes designed affordances rather than classroom uptake. *Meilenstein* already offers a relatively strong basis for sustainability-oriented intercultural competence development, above all in units built around intercultural comparison, communicative participation, and socially relevant themes. This is most visible in the clusters shown in Figure 3, especially those concerned with family, relationships, values, personal development, customs, identity, sustainability, and civic engagement. Teachers can build on these strengths instead of treating sustainability as an external addition to the textbook.

The weaker VJ dimension still calls for pedagogical enrichment. In many units, values are present only implicitly through themes or examples and are not systematically turned into tasks that require sustained reflection, ethical positioning, or critical comparison. Teachers may therefore need to add reflective prompts, writing tasks, structured discussion, or project-based activities in order to deepen learners’ engagement with questions of responsibility, justice, and coexistence. Future textbook design could strengthen this dimension by integrating more tasks that connect intercultural content with evaluation, dilemma-based reflection, or civic participation. A behaviorally informed textbook unit should ideally guide learners through a fuller sequence of learning behaviors: input, comparison, reflection, position-taking, communication, and action proposal. Cultural and sustainability-related texts provide the initial input; comparison tasks help learners identify differences and commonalities; reflection prompts guide learners to examine values and assumptions; position-taking tasks require learners to articulate and justify judgments; communicative tasks create opportunities for negotiation and mediation; and action-oriented projects invite learners to propose context-sensitive responses (Kolb, 1984; Nguyen, 2025).

The thematic differences identified in Figure 3 also show that not all unit types offer the same pedagogical potential. Everyday communicative units are particularly strong in IA but often weaker in VJ. By contrast, units dealing with family, identity, sustainability, and social responsibility tend to provide more balanced support across all three dimensions. This means that sustainability-oriented intercultural competence development in foreign-language education may depend less on isolated “sustainability units” than on how different thematic domains are pedagogically connected across the textbook sequence.

A more specific implication follows from the focal-unit analysis. If teachers aim to strengthen sustainability-oriented intercultural competence development rather than only intercultural knowledge or communicative fluency, they may need to intervene at the level of task design. In many units, including some of the more reflective higher-level units, the textbook already offers strong thematic input, but the value-judgment or action-oriented dimensions remain underdeveloped because the follow-up activities stop at description, comparison, or opinion exchange. Additional prompts that require learners to weigh competing values, address inequality or responsibility, or formulate action-relevant responses could therefore strengthen the pedagogical contribution of otherwise strong units without changing their thematic focus.

### 4.5. Limitations and Further Research

Several limitations should be acknowledged. First, the study analyzes textbook-embedded, design-level learning-behavioral affordances rather than learners’ actual classroom behavior. It also does not validate “sustainable intercultural competence” as a new psychometric construct but uses the term only as a working shorthand for the sustainability-oriented development of intercultural competence. Therefore, the findings show what kinds of learning behaviors are pedagogically invited by the textbook, but they do not demonstrate how learners actually perceive, take up, or enact these opportunities in classroom practice (Nguyen, 2025). Second, the study focuses on one recently published German textbook series designed for university-level German-major instruction in China and therefore does not claim to represent all German-language teaching materials. Third, the coding was conducted at the unit level in order to identify broader pedagogical patterns across 37 units. This makes the analysis suitable for textbook-wide comparison, but it does not provide a full page-by-page or task-by-task micro-analysis of all materials. Fourth, although the coding was strengthened through independent checking and consensus discussion, formal statistical intercoder reliability was not calculated. This should be considered when interpreting the numerical aggregation of the 0–2 scores. Fifth, although the study combines qualitative interpretation with descriptive frequency comparison, its primary aim remains interpretive rather than statistical. The scores therefore function as analytical supports for pattern identification rather than as claims of exact measurement.

These limitations also point to directions for further research. Future studies could compare *Meilenstein* with other German textbook series used in China, examine how teachers actually mediate value-oriented dimensions in classroom practice, or conduct more fine-grained task-level analyses of selected units. It would also be valuable to investigate whether learners themselves perceive and enact the same affordance imbalance identified in the present textbook analysis, especially between communicative interaction and reflective value judgment. Future research could combine textbook analysis with classroom observation, learner interviews, reflective journals, task-performance data, or behavioral indicators. Recent behavioral sciences research shows that different data sources, such as behavioral indicators and verbal reports, may capture different aspects of language-related processing and evaluation; a similar multi-method approach could clarify how textbook-designed affordances become actual learning behaviors (Huang & Zhang, 2026).

## 5. Conclusions

This study examined how *Meilenstein*, a recently published German textbook series designed for university-level German-major instruction in China, provides textbook-embedded learning-behavioral affordances for sustainability-oriented intercultural competence development. It did not treat “sustainable intercultural competence” as a standardized independent construct. Instead, drawing on Klieme’s competence-model logic, it developed a three-dimensional analytical framework that translates sustainability-oriented intercultural learning aims into cognitive-understanding, reflective value-judgment, and interaction-action affordance dimensions.

The findings show that *Meilenstein* provides substantial but uneven affordances across the four volumes. CU and IA affordances are consistently stronger than reflective VJ affordances, which indicates that the series is more successful in supporting intercultural understanding and communicative engagement than in scaffolding explicit evaluative reflection. The most balanced affordance structures appear in units dealing with family, relationships, values, identity, sustainability, and civic engagement, whereas everyday practical units privilege IA and later disciplinary units privilege CU. Theoretically, the study contributes by linking Klieme’s competence-model logic with affordance-oriented textbook analysis. This combination shows how broad educational aims such as sustainability-oriented intercultural learning can be translated into operational textbook-level indicators. It also demonstrates that the key issue in textbook-based intercultural competence development is not only what cultural or sustainability-related content is represented but how such content is organized as opportunities for knowing, judging, and acting. From a pedagogical perspective, *Meilenstein* offers a useful basis for sustainability-oriented intercultural learning, but the weaker VJ dimension calls for further enrichment. On the basis of the textbook analysis, teachers may need to add reflective prompts, structured discussion, writing tasks, or project-based work that brings questions of responsibility, justice, coexistence, and evaluation more clearly into view. Future textbook design could strengthen this dimension by integrating more tasks that require ethical positioning, dilemma-based reflection, and civic participation. Overall, the study underscores the value of treating textbooks not only as language-learning materials but also as pedagogical environments in which intercultural knowledge, social values, and communicative action are organized as learning-behavioral opportunities.

## Figures and Tables

**Table 1:** Operational indicators for textbook-embedded learning-behavioral affordances.

Dimension	Main Coding Question	Typical Indicators
Cognitive-understanding affordance (CU)	Does the material invite learners to understand, compare, or interpret cultural, social, or sustainability-related issues?	Cultural difference; communicative norms; festivals and customs; social practices; global issues; intercultural perspectives
Reflective value-judgment affordance (VJ)	Does the material invite learners to evaluate, reflect, or take an ethical position?	Responsibility; equality; respect; openness; stereotypes; media use; sustainability reflection; future-oriented coexistence
Interaction-action affordance (IA)	Does the material invite learners to express, negotiate, mediate, propose, or participate in problem-oriented communication?	Discussion; interview; presentation; mediation; comparison; suggestions; solution-oriented tasks

Note: The indicators were used as operational guides rather than as mechanically exhaustive criteria. In line with Klieme’s competence-model logic, they translate broad educational aims into analyzable textbook-level indicators, but they do not measure learners’ actual competence.

**Table 2:** Score differentiation and sample coding decisions for the three affordance dimensions.

Dimension	Score 0	Score 1	Score 2
Cognitive-understanding affordance (CU)	No meaningful cultural, social, or sustainability-related issue is introduced.	A relevant issue is present, but it is mentioned briefly, implicitly, or without sustained comparison or interpretation.	The unit explicitly and repeatedly invites learners to identify, compare, explain, or interpret cultural, social, or sustainability-related meanings.
Reflective value-judgment affordance (VJ)	No reflective or evaluative positioning is required.	Values or responsibility are implied by the topic, but learners are not systematically guided to evaluate, justify, or reflect.	Learners are explicitly invited to examine assumptions, articulate values, justify positions, or reflect on responsibility, inequality, environmental consequences, or future-oriented coexistence.
Interaction-action affordance (IA)	No communicative or problem-oriented action is required beyond reception or controlled practice.	Learners are invited to exchange information or opinions, but the task remains limited, isolated, or weakly problem-oriented.	Learners are explicitly invited to discuss, negotiate, mediate, propose solutions, roleplay, interview, or participate in communicative problem-solving.

Note: The examples are paraphrased coding rationales rather than verbatim textbook excerpts. Detailed sample coding decisions, including representative unit-level examples, are provided in Table S6.

## Data Availability

The original contributions presented in this study are included in the article and Supplementary Materials. Further inquiries can be directed to the corresponding author.

## Supplementary Materials

The following supporting information can be downloaded at: https://www.mdpi.com/article/10.3390/bs16061028/s1, Table S1: Unit-by-unit coding summary of *Meilenstein*; Table S2: Volume-level summary of the three affordance dimensions; Table S3: Thematic-cluster summary of the three affordance dimensions; Table S4: Overview of selected focal units; Table S5: Fine-grained coding of selected focal units; Table S6: Detailed sample coding decisions and score differentiation.

## Abbreviations

ESD	Education for Sustainable Development
LOTEs	Languages other than English
BNE	Bildung für nachhaltige Entwicklung
DaF	Deutsch als Fremdsprache
DaZ	Deutsch als Zweitsprache
CU	Cognitive-understanding affordance
VJ	Reflective value-judgment affordance
IA	Interaction-action affordance

## References

[B1-behavsci-16-01028] Aronin L., Singleton D. (2012). Affordances theory in multilingualism studies. Studies in Second Language Learning and Teaching.

[B2-behavsci-16-01028] Baker W. (2015). Research into practice: Cultural and intercultural awareness. Language Teaching.

[B3-behavsci-16-01028] Baker W. (2022). Intercultural and transcultural awareness in language teaching.

[B4-behavsci-16-01028] Bandura A. (1986). Social foundations of thought and action: A social cognitive theory.

[B5-behavsci-16-01028] Beltrán-Véliz J. C., Gálvez-Nieto J. L., Klenner-Loebel M., Vera-Gajardo N. (2024). Adaptation and validation of the Intercultural Effectiveness Scale in a sample of initial teacher training students in Chile. Behavioral Sciences.

[B6-behavsci-16-01028] Bolten J. (2001). Interkulturelles coaching, mediation, training und consulting als aufgaben des personalmanagements internationaler unternehmen. Strategisches personalmanagement in globalen unternehmen.

[B7-behavsci-16-01028] Bolten J. (2016). Interkulturelle kompetenz: Eine ganzheitliche perspektive. Polylog. Zeitschrift für interkulturelles Philosophieren.

[B8-behavsci-16-01028] Bolten J. (2018). Einführung in die interkulturelle wirtschaftskommunikation.

[B9-behavsci-16-01028] Borghetti C. (2017). Is there really a need for assessing intercultural competence? Some ethical issues. Journal of Intercultural Communication.

[B10-behavsci-16-01028] Budde J., Blasse N. (2023). Bildung für nachhaltige entwicklung zwischen programmatik und praxis. ZEP—Zeitschrift für Internationale Bildungsforschung und Entwicklungspädagogik.

[B11-behavsci-16-01028] Byram M. (1997). Teaching and assessing intercultural communicative competence.

[B12-behavsci-16-01028] Byram M., Wagner M. (2018). Making a difference: Language teaching for intercultural and international dialogue. Foreign Language Annals.

[B13-behavsci-16-01028] Canale G. (2021). The language textbook: Representation, interaction and learning. Language, Culture and Curriculum.

[B14-behavsci-16-01028] Dawe G., Jucker R., Martin S. (2005). Sustainable development in higher education: Current practice and future developments.

[B15-behavsci-16-01028] Elo S., Kyngäs H. (2008). The qualitative content analysis process. Journal of Advanced Nursing.

[B16-behavsci-16-01028] Feike J. (2019). Kulturelles lernen im DaF/DaZ-unterricht: Paradigmenwechsel in der landeskunde. Zeitschrift für Interkulturellen Fremdsprachenunterricht.

[B17-behavsci-16-01028] Ge N. (2022). Cultural representation in China’s German textbooks: Taking studienweg deutsch as an example. Foreign Language Education in China.

[B18-behavsci-16-01028] Ge N., Wang E., Li Y. (2023). Foreign language education for sustainable development in China: A case study of German language education. Sustainability.

[B19-behavsci-16-01028] González-Salamanca J. C., Agudelo O. L., Salinas J. (2020). Key competences, education for sustainable development and strategies for the development of 21st century skills: A systematic literature review. Sustainability.

[B20-behavsci-16-01028] Hsieh H. F., Shannon S. E. (2005). Three approaches to qualitative content analysis. Qualitative Health Research.

[B21-behavsci-16-01028] Huang X., Zhang X. (2026). Behavioral indicators and verbal judgments in the perception of translation quality: A cognitive experimental study. Behavioral Sciences.

[B22-behavsci-16-01028] Kapranov O. (2021). Discursive representations of education for sustainable development in policy documents by English medium instruction schools in Estonia and Norway. Discourse and Communication for Sustainable Education.

[B23-behavsci-16-01028] Klieme E., Avenarius H., Blum W., Döbrich P., Gruber H., Prenzel M., Reiss K., Riquarts K., Rost J., Tenorth H.-E., Vollmer H. J. (2003). Zur entwicklung nationaler bildungsstandards: Eine expertise.

[B24-behavsci-16-01028] Knapp K., Bolten J. (1995). Interkulturelle kommunikationsfähigkeit als qualifikationsmerkmal in der Wirtschaft. Cross culture: Interkulturelles handeln in der wirtschaft.

[B25-behavsci-16-01028] Kolb D. A. (1984). Experiential learning: Experience as the source of learning and development.

[B26-behavsci-16-01028] Kong D., Jia W. (2025). Meilenstein *[新经典德语]*.

[B27-behavsci-16-01028] Kramsch C. (1993). Context and culture in language teaching.

[B28-behavsci-16-01028] Krippendorff K. (2018). Content analysis: An introduction to its methodology.

[B29-behavsci-16-01028] Li J., Jia W. (2023). Meilenstein *[新经典德语]*.

[B30-behavsci-16-01028] Li Y., Jia W. (2025). Meilenstein *[新经典德语]*.

[B31-behavsci-16-01028] Li Y., Hua Y. (2025). The shift towards sustainable development in foreign language education and its implications: A case study of Germany. Foreign Language Education & Research.

[B32-behavsci-16-01028] Liu H. (2003). Intercultural communicative competence and its cultivation: A constructivist perspective. Foreign Languages and Teaching.

[B33-behavsci-16-01028] Mayring P. (2000). Qualitative content analysis. Forum: Qualitative Social Research.

[B34-behavsci-16-01028] Monteiro M. J. P. (2007). Deutsche Fachsprachen: Alte und neue Erfahrungen mit dem Begriff “Kultur” in den Geisteswissenschaften und im Bereich Deutsch als Fremdsprache. Pandaemonium Germanicum.

[B35-behavsci-16-01028] Müller D. (2022). Nachhaltigkeit im Fremdsprachenlernen: Ansätze für den universitären DaF-Unterricht unter der Zielsetzung einer Bildung für nachhaltige Entwicklung (BNE). Informationen Deutsch als Fremdsprache.

[B36-behavsci-16-01028] Müller S., Gelbrich K. (2004). Interkulturelles marketing.

[B37-behavsci-16-01028] Nguyen Q. N. (2025). Affordance theory in language education: A multidimensional framework. TESL-EJ.

[B38-behavsci-16-01028] Pan Y. (2008). Eine empirische Studie zur Förderung der interkulturellen kompetenzen von studenten mit fremdsprachen als hauptfach in China. China Foreign Languages.

[B39-behavsci-16-01028] Ping X., Wang J. (2023). A study on the representation of global competence in English textbooks for higher vocational education in China. SAGE Open.

[B40-behavsci-16-01028] Pinto P. T. (2021). The sustainable development goals (SDGs) words “poverty” and “sustainability” in the Brazilian research. Cadernos de Linguística.

[B41-behavsci-16-01028] Risager K., Preisler B., Fabricius A., Haberland H., Kjærbeck S., Risager K. (2005). Languaculture as a key concept in language and culture teaching. The consequences of mobility.

[B42-behavsci-16-01028] Sachdev K. K., Vibulphol J. (2025). Promoting sustainability through language learning: Analysing English textbooks in Thailand. International Education Studies.

[B43-behavsci-16-01028] Schreiber J.-R., Siege H. (2016). Orientierungsrahmen für den lernbereich globale entwicklung.

[B44-behavsci-16-01028] Solomon B. D. (2023). World Commission on Environment and Development (WCED). Dictionary of ecological economics: Terms for the new millennium.

[B45-behavsci-16-01028] Stibbe A. (2008). Words and worlds: New directions for sustainability literacy. Language and Ecology.

[B46-behavsci-16-01028] Svalfors U. (2017). Education for sustainable development and multidimensional implementation: A study of implementations of sustainable development in education with the curriculum of upper secondary school in Sweden as an example. Discourse and Communication for Sustainable Education.

[B47-behavsci-16-01028] Tang L., Zhang C. (2023). Intercultural friendships with international students in China: Examining the role of intergroup contact, intercultural communication competence, host country nationals’ attitudes, and perceived intergroup threats. Behavioral Sciences.

[B48-behavsci-16-01028] UNESCO (2018). Issues and trends in education for sustainable development.

[B49-behavsci-16-01028] Vare P., Scott W. (2007). Learning for a change: Exploring the relationship between education and sustainable development. Journal of Education for Sustainable Development.

[B50-behavsci-16-01028] Wals A. E. J., Kieft G. (2010). Education for sustainable development: Research overview.

[B51-behavsci-16-01028] Weiss M., Barth M. (2020). Case survey analysis of sustainability curricula in higher education. Work Papers on Higher Education Studies in Sustainable Development.

[B52-behavsci-16-01028] Yang L., Wang H., Zhang H., Long H. (2024). The relationships of self-sustained English learning, language mindset, intercultural communicative skills, and positive L2 self: A structural equation modeling mediation analysis. Behavioral Sciences.

[B53-behavsci-16-01028] Zhan X., Jia W. (2024). Meilenstein *[新经典德语]*.

[B54-behavsci-16-01028] Zygmunt T. (2016). Language education for sustainable development. Discourse and Communication for Sustainable Education.

