# Supplementary material for: Learning-Behavioral Affordances in German Textbooks: Sustainability-Oriented Intercultural Competence Development in China
Source: Behav Sci (Basel). 2026 Jun 19;16(6):1028. doi: 10.3390/bs16061028 (PMC13296004; doi:10.3390/bs16061028)
Supplement: Supplementary file 1 [file behavsci-16-01028-s001.zip › behavsci-4363536-supplementary.pdf]

Overview of supplementary materials

The supplementary materials provide the coding evidence and supporting documentation for the main analysis. Table 1 in the main manuscript defines the operational indicators for the three affordance dimensions, while Table 2 clarifies the 0–2 score differentiation criteria. Building on these two tables, Table S1 provides the full unit-by-unit coding results for all 37 units of Meilenstein. Tables S2 and S3 summarize these unit-level scores by volume and thematic cluster, corresponding to the aggregate patterns visualized in Figures 2 and 3 in the main manuscript. Tables S4 and S5 provide a closer examination of selected focal units discussed in Section 3.4. Finally, Table S6 presents representative sample coding decisions, paraphrased evidence, and coding rationales to illustrate how the scoring criteria were applied in practice. Together, these materials make the analytical process more transparent and allow readers to trace the connection between operational indicators, coding decisions, aggregate findings, and interpretive claims.

Table S1 provides a condensed unit-level coding overview of all 37 units and serves as the coding index underlying the summary patterns reported in Figure 2 and Figure 3.

Table S1. Unit-by-Unit Coding Summary of Meilenstein

Volume	Unit	Main Topic	Cog.-Und.	Val.-Judg.	Int.-Act.
1	L1	University life / German studies / campus	1	0	1
1	L2	Transport / directions / travel options	1	0	2
1	L3	Daily routine / free time / appointments	1	0	2
1	L4	Shopping / consumption / wishes	1	1	2
1	L5	Health / symptoms / advice	2	1	2
1	L6	Housing / living space / rules	1	1	2
1	L7	Food / restaurant / eating habits	2	1	2
1	L8	Gifts / congratulations / celebrations	2	2	2
1	L9	Festivals / customs / New Year	2	2	1
2	L1	Family meanings and family relations	2	2	2
2	L2	Appearance / partner preferences	1	1	2
2	L3	Looking back / looking ahead	1	1	2
2	L4	Distance / travel / mobility	1	0	2
2	L5	Living environments / where to live better	2	1	2
2	L6	Customs in different countries	2	2	2
2	L7	Future plans	1	1	2
2	L8	Career choice / ability and aspiration	1	1	2
2	L9	Job application	1	0	2
2	L10	Media importance	1	1	2
2	L11	Internet use across age groups	1	1	2

2	L12	Life without smartphones	1	2	2
3	L1	Happiness / childhood / well-being	2	1	2
3	L2	Heroes and role models	2	2	2
3	L3	Politeness in German	2	2	2
3	L4	Language change	2	0	2
3	L5	Climate change and environmental protection	2	2	2
3	L6	Sustainability	2	2	2
3	L7	Globally connected world	2	1	2
3	L8	Exchange and engagement	2	2	2
4	L1	Youth values	2	2	2
4	L2	Individual and social responsibility	2	2	2
4	L3	Heimat and identity	2	2	2
4	L4	Exchange and change	2	2	2
4	L5	Visual representation and description / art	2	1	2
4	L6	Literature and interpretation	2	1	1
4	L7	Knowledge and science	2	0	1
4	L8	Language research and language learning	2	0	1

Table S2. Distribution of Sustainable Intercultural Competence across the Four Volumes of *Meilenstein*

Volume	No. of Units	Cog.-Und. (Total)	Val.-Judg. (Total)	Int.-Act. (Total)	Predominant Orientation	Representative Units
Vol. 1	9	13	7	16	Interaction-oriented everyday intercultural learning	L5, L7, L8, L9
Vol. 2	12	15	13	24	Strong communicative orientation with growing social-value concerns	L1, L5, L6, L12
Vol. 3	8	16	12	16	Most balanced volume, with clear sustainability-related expansion	L1, L5, L6, L8
Vol. 4	8	16	11	14	Abstract, reflective, and knowledge-based intercultural development	L1, L2, L3, L4
Total	37	60	43	70	Cognitive-understanding and interaction-action stronger than value-judgment overall	-

Table S3. Thematic Patterns of Sustainable Intercultural Competence in *Meilenstein*

Unit Cluster	No. of Units	Cog.-Und.	Val.-Judg.	Int.-Act.	Main Pattern
Everyday life, mobility, and practical situations	8	11	5	16	Strong in communicative practice; value reflection mostly weak or implicit
Family, relationships, values, and personal development	9	14	13	18	Strongest cluster for linking everyday experience, values, and self-reflection
Customs, identity, and intercultural exchange	5	10	10	9	Particularly rich in cultural comparison and implicit value orientation
Media, digitalization, and global connectivity	4	5	5	8	Emphasizes discussion and social relevance, but often with moderate depth
Sustainability, environment, and civic participation	3	6	6	6	Most explicit sustainability-oriented cluster; relatively balanced across dimensions
Language, literature, arts, and knowledge	8	14	4	13	Strong in cognitive and communicative dimensions, but weaker in explicit value reflection

Table S4. Summary of Fine-Grained Coding across the Eight Focal Units

Unit	Texts mainly support	Tasks mainly support	Reflection prompts mainly support	Main imbalance / distinctive feature
Vol. 2, L1 Family meanings and family relations	cognitive-understandin g + value-judgment	interaction-action + reflective comparison	value-judgment	Action remains comparatively discursive
Vol. 2, L6 Customs in different countries	cognitive-understandin g + value-judgment	interaction-action + strategic communication	critical value reflection	Comparatively balanced
Vol. 2, L12 Life without smartphones	cognitive-understandin g + value-judgment	interaction-action through argumentation	responsible self-positioning	Action remains largely discursive
Vol .3, L1 Happiness / childhood / well-being	cognitive-understandin g	interaction-action through narration and interviewing	moderate value reflection	Value-judgment comparatively weaker
Vol. 3, L5 Climate change and environmental protection	cognitive-understandin g + global problem framing	interaction-action through solution-oriented tasks	responsibility and commitment	Comparatively balanced
Vol. 3, L6 Sustainability	cognitive-understandin g + value-judgment	interaction-action through proposal-making	cultural and ethical reflection	Highly integrated
Vol. 3, L8 Exchange and engagement	global citizenship knowledge + value orientation	interaction-action through mediation and engagement	reflective communication	One of the strongest integrations
Vol. 4, L2/L3 Responsibility / identity	cognitive-understandin g + value-judgment	reflective interaction	higher-order self-positioning	Action less concrete than in explicit sustainability units

Table S5. Text-/Task-Level Coding of Selected Focal Units

Volume	Unit	Item	Type	Cog.-Und.	Val.-Judg.	Int.-Act.	Main Function
2	L1	Statistical text on family structures in Germany (2022 vs. 1996)	Text	2	1	0	socio-cultural knowledge
2	L1	Lexical-semantic cluster: Vertrauen, Verständnis, Partnerschaft, Verantwortung, Unterstützung	Text	1	2	0	value orientation
2	L1	Discussing the meaning of family	Task	1	2	2	reflective discussion
2	L1	Explaining, comparing, and evaluating life and family forms	Task	2	2	2	intercultural comparison
2	L1	Prompt: family as love, trust, support, and happiness	Reflection prompt	1	2	1	value reflection
2	L6	Typical gestures in different countries and their meanings	Text	2	1	0	intercultural knowledge
2	L6	Rules for forms of address, invitations, and taboos in different countries	Text	2	2	0	norm awareness
2	L6	Reporting intercultural experiences	Task	1	1	2	experience narration
2	L6	Developing intercultural strategies	Task	1	2	2	strategic communication
2	L6	Discussing and reflecting on national stereotypes	Reflection prompt	1	2	2	critical reflection
2	L12	Functions of smartphones and digital devices in everyday life	Text	1	1	0	everyday digital literacy
2	L12	Pros and cons of smartphone use	Text	1	2	0	evaluative framing
2	L12	Discussing the role of smartphones in everyday life	Task	1	1	2	communicative discussion
2	L12	Arguing for or against smartphone use	Task	1	2	2	argumentation
2	L12	Reflecting on the proper use of smartphones	Reflection prompt	1	2	1	responsible self-positioning
3	L1	Cross-generational and cross-cultural perspectives on childhood	Text	2	1	0	intercultural comparison
3	L1	Happiness-related statements linking well-being to family, health, education, and social	Text	2	1	0	social meaning of happiness

		relations					
3	L1	Talking about one's own childhood	Task	1	1	2	experience narration
3	L1	Conducting an interview on happiness and reporting results	Task	1	1	2	interview/reporting
3	L1	Comparing childhood across generations and cultures	Reflection prompt	2	1	2	comparison and interpretation
3	L5	Causes and dangers of climate change	Text	2	1	0	issue knowledge
3	L5	Extreme weather cases	Text	2	2	0	global problem awareness
3	L5	Expressing ideas about future cities	Task	1	1	2	future-oriented discussion
3	L5	Formulating concrete measures for climate protection and adaptation	Task	1	2	2	action-oriented communication
3	L5	Setting personal climate and environmental goals / understanding global responsibility	Reflection prompt	1	2	2	responsibility and commitment
3	L6	Global problems: pollution, inequality, disease, conflict, poverty	Text	2	2	0	global issue awareness
3	L6	Three-pillar model / sustainability triangle	Text	2	1	0	conceptual understanding
3	L6	Collecting measures to promote sustainability	Task	1	2	2	problem-solving discussion
3	L6	Making suggestions for a sustainable everyday life	Task	1	2	2	everyday action orientation
3	L6	Presenting sustainable cultural heritage sites	Reflection prompt	2	1	2	cultural sustainability
3	L8	International organisations and their tasks	Text	2	2	0	global citizenship knowledge
3	L8	Social engagement and the meaning of civic participation	Text	1	2	0	social responsibility
3	L8	Gathering and passing on information about study and internship abroad	Task	1	1	2	communicative mediation
3	L8	Learning UN-related competences and building a professional profile	Task	2	2	2	competence-based action orientation
3	L8	Reporting on everyday life, study, and engagement	Reflection prompt	1	1	2	reflective communication

4	L2	Core theme: improving the world and closing gaps in social coexistence	Text	2	2	0	ethical-social framing
4	L2	Fair business practices and support for community development	Text	2	2	0	social ethics
4	L2	Discussing what social responsibility means in everyday life	Task	1	2	2	critical discussion
4	L2	Evaluative exercises on responsibility-related statements	Task	1	2	1	evaluative interpretation
4	L2	Reflection on one's own contribution to a better future	Reflection prompt	1	2	2	personal-social positioning
4	L3	Speaking about Heimat and identity	Text	2	1	0	conceptual-cultural understanding
4	L3	Reflecting on the individual and the collective	Text	2	2	0	identity and belonging
4	L3	Discussing Heimat and identity	Task	1	2	2	reflective interaction
4	L3	Analysing literary texts and writing summaries	Task	2	1	1	interpretive literacy
4	L3	Reflection on belonging, memory, and collective identity	Reflection prompt	1	2	1	higher-order value reflection

Table S6. Detailed sample coding decisions and score differentiation

No.	Unit / Lesson	Dimension	Score	Evidence and Coding Rationale
1	Vol. 1, Lektion 2, "Entschuldigung, wie komme ich zum Bahnhof?"	CU	1	**Evidence**: The unit introduces everyday sociocultural situations such as asking for directions, understanding transport-related information, and handling mobility-related communication. **Rationale**: The unit provides limited cultural or social contextualization, but the main purpose is practical communication. Cultural interpretation or sustained comparison is present only implicitly, so CU is coded as 1.
2	Vol. 1, Lektion 2, "Entschuldigung,	VJ	0	**Evidence**: The tasks focus on functional language use in daily mobility situations and do not ask

No.	Unit / Lesson	Dimension	Score	Evidence and Coding Rationale
	wie komme ich zum Bahnhof?”			learners to evaluate values, responsibilities, social assumptions, or ethical positions. **Rationale**: No clear reflective or evaluative positioning is required. The unit may support communication, but it does not scaffold value judgment; therefore, VJ is coded as 0.
3	Vol. 1, Lektion 2, “Entschuldigung, wie komme ich zum Bahnhof?”	IA	2	**Evidence**: Learners are invited to use German in simulated daily-life situations, such as asking for directions, understanding routes, and completing mobility-related communicative tasks. **Rationale**: The unit explicitly supports communicative participation and action-oriented language use. Because learners are repeatedly invited to act through German in practical contexts, IA is coded as 2.
4	Vol. 1, Lektion 9, “Frohes neues Jahr!”	CU	2	**Evidence**: The unit introduces festivals, holidays, customs, and culturally embedded practices related to New Year and Spring Festival contexts. **Rationale**: The material explicitly invites learners to notice, compare, and interpret cultural practices. Because cultural understanding is developed beyond isolated vocabulary or facts, CU is coded as 2.
5	Vol. 1, Lektion 9, “Frohes neues Jahr!”	VJ	1	**Evidence**: The unit may invite learners to compare festivals, customs, or celebratory practices, but it does not consistently require them to examine assumptions, evaluate values, or justify ethical positions. **Rationale**: Value orientation is present but weakly scaffolded. Learners may notice difference, but they are not systematically guided toward reflective judgment; therefore, VJ is coded as 1.
6	Vol. 2, Lektion 11, “Nie zu alt fürs Internet!”	CU	2	**Evidence**: The unit introduces digital media use, internet-related practices, media competence, and intergenerational differences in digital communication. **Rationale**: The unit explicitly supports understanding of contemporary social and communicative practices. Learners are invited to interpret digital communication as a social and

No.	Unit / Lesson	Dimension	Score	Evidence and Coding Rationale
				cultural phenomenon, so CU is coded as 2.
7	Vol. 2, Lektion 11, “Nie zu alt fürs Internet!”	VJ	1	**Evidence**: The topic implies responsibility, inclusion, generational difference, media participation, and digital competence, but reflection depends on whether discussion tasks are extended into explicit evaluation. **Rationale**: The unit contains value-relevant potential, but the reflective dimension is not always systematically scaffolded. Because responsibility and digital inclusion are implied more than explicitly developed, VJ is coded as 1.
8	Vol. 2, Lektion 11, “Nie zu alt fürs Internet!”	IA	2	**Evidence**: Learners are invited to discuss internet use, media practices, generational experiences, and digital participation in German. **Rationale**: The unit provides repeated opportunities for communicative participation and opinion exchange around a real-world social topic. IA is therefore coded as 2.
9	Vol. 3, Lektion 5, “Klimawandel und Umweltschutz”	CU	2	**Evidence**: The unit introduces climate change, environmental protection, climate and environmental signs, and possible forms of action or adaptation. **Rationale**: Sustainability-related concepts are explicitly introduced and developed through thematic input. Learners are invited to understand and interpret ecological and social meanings, so CU is coded as 2.
10	Vol. 3, Lektion 5, “Klimawandel und Umweltschutz”	VJ	2	**Evidence:** Learners are invited to consider environmental responsibility, consequences of climate change, and possible responses to ecological problems. **Rationale:** The unit explicitly requires reflection on responsibility, consequences, and future-oriented coexistence. Because value judgment is directly scaffolded, VJ is coded as 2.
11	Vol. 3, Lektion 5, “Klimawandel und Umweltschutz”	IA	2	**Evidence:** Learners discuss environmental issues, communicate about possible responses, and formulate ideas related to environmental protection or problem-solving. **Rationale:** The unit invites learners to use German to discuss real-world problems and propose or negotiate responses. This

No.	Unit / Lesson	Dimension	Score	Evidence and Coding Rationale
				sustained communicative and problem-oriented design supports IA = 2.
12	Vol. 3, Lektion 6, “Rund um die Nachhaltigkeit”	CU	2	**Evidence:** The unit connects global problems, the importance of sustainability, future-oriented questions, and sustainable ways of living. **Rationale:** Sustainability is not treated only as an isolated topic but is connected to everyday life, global responsibility, and social practice. The unit therefore provides strong CU affordance and is coded as 2.
13	Vol. 3, Lektion 6, “Rund um die Nachhaltigkeit”	VJ	2	**Evidence:** Learners are invited to consider sustainability, responsibility, global problems, and the consequences of individual or collective actions. **Rationale:** The unit explicitly encourages learners to evaluate responsibility and connect personal choices with broader sustainability concerns. VJ is therefore coded as 2.
14	Vol. 3, Lektion 6, “Rund um die Nachhaltigkeit”	IA	2	**Evidence:** Learners discuss sustainability-related practices and may propose suggestions or responses connected to daily life and future-oriented action. **Rationale:** The unit moves beyond comprehension by inviting learners to communicate, discuss, and propose action-oriented responses. IA is therefore coded as 2.
15	Vol. 4, Lektion 2, “Individuum und soziale Verantwortung”	CU	2	**Evidence:** The unit introduces individual responsibility, social responsibility, personal development, moral action, reliability, leadership, and social engagement. **Rationale:** Responsibility is explicitly developed as a social and personal concept. Learners are invited to understand and interpret responsibility-related meanings, so CU is coded as 2.
16	Vol. 4, Lektion 2, “Individuum und soziale Verantwortung”	VJ	2	**Evidence:** Learners are invited to consider what responsible action means in personal, social, or collective life. **Rationale:** The unit directly scaffolds value reflection and ethical self-positioning. Because learners are asked to reflect on responsibility and social action, VJ is coded as 2.
17	Vol. 4, Lektion 2, “Individuum und soziale	IA	1	**Evidence:** Learners may discuss responsibility and social engagement, but communicative tasks are less concretely problem-solving-oriented than in the

No.	Unit / Lesson	Dimension	Score	Evidence and Coding Rationale
	Verantwortung"			climate-change unit. **Rationale:** IA is present through discussion, but it is less sustained and less action-oriented than in units requiring concrete proposals or problem-solving. IA is therefore coded as 1.
18	Vol. 4, Lektion 3, "Heimat und Identität"	CU	2	**Evidence:** The unit addresses belonging, cultural affiliation, identity, migration-related experience, and self-positioning in culturally diverse contexts. **Rationale:** The unit strongly supports interpretation of complex cultural and social meanings. Learners are invited to understand identity and belonging beyond factual cultural description, so CU is coded as 2.
19	Vol. 4, Lektion 3, "Heimat und Identität"	VJ	2	**Evidence:** Learners are invited to reflect on belonging, identity, cultural affiliation, and their own position in relation to culturally diverse experience. **Rationale:** The unit requires reflective self-positioning and evaluation of identity-related meanings. Because value judgment is directly connected to self-reflection, VJ is coded as 2.
20	Vol. 4, Lektion 3, "Heimat und Identität"	IA	1	**Evidence:** The unit offers opportunities for discussion of identity and belonging, but the tasks are more reflective than solution-oriented or action-oriented. **Rationale:** Learners may exchange views, but the unit does not consistently require negotiation, mediation, or problem-oriented action. IA is therefore coded as 1.
21	Vol. 4, Lektion 6, "Dichtung und Deutung"	CU	2	**Evidence:** The unit introduces literary knowledge, literary interpretation, the relation between literature and cultural exchange, and the question of literature in a globalized context. **Rationale:** The unit strongly supports cognitive and interpretive understanding. Because learners engage with complex literary, cultural, and disciplinary meanings, CU is coded as 2.
22	Vol. 4, Lektion 6, "Dichtung und Deutung"	VJ	1	**Evidence:** The unit may encourage interpretation of literary and cultural content, but it does not consistently require ethical evaluation, social responsibility, or future-oriented reflection. **Rationale:** Value positioning is present only indirectly through interpretation and

No.	Unit / Lesson	Dimension	Score	Evidence and Coding Rationale
				perspective-taking. Because explicit reflective value judgment is limited, VJ is coded as 1.
23	Vol. 4, Lektion 6, "Dichtung und Deutung"	IA	1	**Evidence:** Learners may discuss literary texts, interpret meanings, and communicate about literature and cultural exchange. **Rationale:** Communication is present, but it remains mainly interpretive or presentational rather than negotiation-based, mediation-based, or problem-solving-oriented. IA is therefore coded as 1.
24	Vol. 2, Lektion 9, "Fit für die Bewerbung!"	VJ	0	**Evidence:** The unit focuses mainly on functional communication, job application preparation, writing an application, understanding job advertisements, and preparing for interview-related situations. **Rationale:** Although the unit may support CU or IA, it does not provide a meaningful opportunity for reflective value judgment concerning responsibility, inequality, ethical positioning, or future-oriented coexistence. VJ is therefore coded as 0.

Note. This table provides representative sample coding decisions used to differentiate scores of 0, 1, and 2 across the three affordance dimensions. The selected units were chosen purposively to cover different volumes, thematic clusters, affordance dimensions, score levels, and both strong and boundary cases. The evidence is paraphrased rather than reproduced verbatim from the textbook. CU = cognitive-understanding affordance; VJ = reflective value-judgment affordance; IA = interaction-action affordance.
